# The prognostic role of preoperative serum albumin levels in glioblastoma patients

**DOI:** 10.1186/s12885-015-1125-0

**Published:** 2015-03-08

**Authors:** Sheng Han, Yanming Huang, Zhonghua Li, Haipei Hou, Anhua Wu

**Affiliations:** Department of Neurosurgery, The First Hospital of China Medical University, Nanjing Street 155, Heping District, Shenyang, 110001 China

**Keywords:** Albumin, Glioblastoma, Prognosis, Nutritional indicator

## Abstract

**Background:**

Serum albumin level is a reliable and convenient marker of the nutritional status of patients, and has been identified as a prognostic marker in glioblastoma. However, because of the recent wide application of standard radio-chemotherapy for the treatment of glioblastoma patients, the prognostic effect of preoperative serum albumin levels needs to be re-evaluated and the related mechanism should be further explored.

**Methods:**

A total of 214 patients with histologically proven glioblastoma who underwent treatment at our institution between 2009 and 2012 were retrospectively analyzed. Clinical information was obtained from electronic medical records. Kaplan–Meier analysis and Cox proportional hazards models were used to examine the survival function of preoperative serum albumin levels in these glioblastoma patients.

**Results:**

Serum albumin levels were significantly correlated with overall survival in glioblastoma patients (multivariate HR = 0.966; 95% CI, 0.938-0.995; *P* = 0.023). Serum albumin level was high in patients receiving standard therapy, which may affect its prognostic significance. Despite the correlation between serum albumin levels and other nutritional indicators such as prealbumin, total protein and total lymphocyte counts, only serum albumin level was an independent predictor of patient survival.

**Conclusions:**

Serum albumin level is associated with prognosis in glioblastoma patients, although the underlying mechanism is complex because of the role of serum albumin as a nutritional indicator and its involvement in inflammatory responses.

## Background

Glioblastoma is the most common malignant primary tumor of the central nervous system. In recent years, surgery combined with radiotherapy and temozolomide (TMZ) chemotherapy has become the standard treatment for glioblastoma patients [1]. However, survival of glioblastoma patients varies significantly even among patients who received the same treatment. This suggests that the survival of glioblastoma patients is influenced by multiple factors, including therapeutic strategies, patient status, and the characteristics of the tumor. Markers related to these factors are generally accepted as prognostic factors for the survival of patients with glioblastoma [2-4]. Serum prognostic factors are of considerable clinical value because of their accessibility.

The prognostic role of nutritional status has been investigated in various cancers. However, recent major clinical studies on glioblastoma did not include consideration of nutritional status as a prognostic factor. The nutritional status of patients can be evaluated by measuring the levels of serum factors such as hemoglobin, insulin-like growth factor-binding protein (IGFBP)-2 or albumin. In our previous study, high serum IGFBP-2 level was related to poor prognosis in glioblastoma patients [5]. High IGFBP-2 levels are significantly associated with low albumin levels [6]. Moreover, hypoalbuminemia is independently associated with poor survival in numerous solid cancers [7]. A relationship between serum albumin and survival in glioblastoma patients has also been reported [8,9]. However, the potential effects of standard therapy and the molecular marker *O*-methylguanine-DNA methyltransferase (MGMT) on the prognostic role of serum albumin remain unclear. In addition, the extent to which the prognostic role of serum albumin is associated with its reflection of nutritional status remains to be determined. In the present study, we retrospectively analyzed 214 patients with glioblastoma treated in our neurosurgical center to examine the prognostic value of preoperative serum albumin levels.

## Methods

### Clinical data acquisition

Patient information, including pathological diagnosis, general condition and biochemistry data (serum albumin, prealbumin, total protein levels and total lymphocyte counts), was collected from the Neurosurgery Department of the First Hospital of China Medical University, Shenyang, over a 4-year period between 2009 and 2012. Patients with other chronic wasting diseases that could influence serum albumin levels or survival or those lacking complete data were excluded. Patients underwent surgical resection by neurosurgeons who used similar operational techniques and principles. Glioblastomas were diagnosed by two neuropathologists according to the World Health Organization 2007 criteria. Overall survival (OS) was defined as the interval between surgery and death from glioblastoma. This study was approved by the institutional review board of The First Hospital of China Medical University, and written informed consent was obtained from each glioma tissue donor, who consented to the use of the tumor tissue and clinical data for future research. The research was in compliance with the Helsinki Declaration.

Serum albumin levels were measured preoperatively. Blood samples were collected in the morning after an overnight fast before medical intervention and were tested by staff at the Department of Clinical Laboratory within 2 h of collection. The normal reference range for serum albumin at our center is 30–50 g/L.

### Adjuvant treatment

Adjuvant treatment consisted of radio-chemotherapy strategies similar to those described by Stupp et al. [1]. The patients who received the whole adjuvant treatment protocol were defined as the “completely applied group” (CAG), and those who did not receive any adjuvant treatment were defined as the “not applied group” (NAG). Patients who did not complete the adjuvant treatment protocol were included in the “partially applied group” (PAG).

### MGMT promoter methylation status

Methylation-specific multiplex ligation-dependent probe amplification (MS-MLPA) was used to evaluate MGMT promoter methylation status in paraffin embedded tumor samples. DNA was extracted from paraffin sections of glioblastoma patients using the Qia-Amp DNA mini kit (Qiagen) after deparaffinization. MS-MLPA was performed using the MS-MLPA probe mix prepared by Salsa MS-MLPA Kit ME011 MMR (MRC-Holland), as described by the manufacturer. After denaturation of the sample, probes were hybridized and then ligated. For half of the sample, ligation was combined with *Hha*I (R6441, Promega) digestion. Agarose gel electrophoresis was used to check MLPA efficiency. PCR was performed, and data were quantified with GeneMarker software (version 1.5, Soft Genetics). The difference in the efficiency of the PCR for the individual samples was normalized by dividing the peak value of each probe by the peak of the control probes. CpGenome Universal Methylated DNA and Unmethylated DNA (Chemicon, Millipore) were included as controls. The methylation ratio was then calculated by dividing each normalized peak value of the digested sample by that of the corresponding undigested sample. The methylation ratio corresponded to the percentage of methylated sequences. A methylation ratio >0.25 was considered as “methylated”, which was consistent with a previous study [10].

### Immunohistochemistry (IHC) for detection of isocitrate dehydrogenase 1 (IDH1) mutation

IDH1 mutation was examined by immunohistochemistry in formalin-fixed and paraffin-embedded tumor samples. Tissue blocks were cut at a thickness of 5-μm. After heat-induced antigen retrieval, sections were incubated with the primary monoclonal IDH1- R132H antibody (clone H09, 1:10 dilution; Dianova, Hamburg, Germany) that specifically recognizes IDH1-R132H mutation status, as previously described [11]. For negative controls, the primary antibody was replaced by normal mouse serum. Diaminobenzidine was used for color development and hematoxylin as counterstain. Results were visualized and photographed under a light microscope (Olympus BX-51; Olympus Optical Co., Ltd., Tokyo, Japan). Two investigators (ZL and HH) evaluated the IHC results. Cases with expression of the mutant IDH1-R132H protein by tumor cells were recorded as positive, and cases without expression of the mutant IDH1-R132H protein by tumor cells were recorded as negative [12].

### Statistical analysis

Cox proportional hazards models were used to calculate hazard ratios (HRs) of death according to the serum albumin levels in glioblastomas, unadjusted and adjusted for sex, age, tumor size, preoperative Karnofsky performance status (KPS), degree of resection, adjuvant treatment, MGMT promoter methylation and IDH1-R132H mutation. To adjust for potential confounders, serum albumin levels, age, tumor size, and preoperative KPS were used as continuous variables and all of the other covariates were used as categorical variables. MGMT promoter methylation status was dichotomized (methylation vs. unmethylation), and IDH1-R132H mutation status was dichotomized (positive vs. negative). Tumor resection was defined as follows: (0) biopsy or subtotal resection with residual tumor ≥30%, (1) subtotal resection with residual tumor <30%, and (2) gross total resection. Adjuvant treatment was defined as described above, namely (0) NAG, (1) PAG, and (2) CAG. In some analyses, serum albumin levels were defined as: (0) <30 g/L, (1) ≥30 g/L; or (0) <30 g/L, (1) 30–40 g/L, (2) ≥40 g/L. Tumor size was calculated based on preoperative MRI scans as follows: longest diameter × widest diameter × thickness (section thickness × the number of layers) × 1/2. Kaplan–Meier survival analysis was used to determine the distribution of OS time, and the results were analyzed with the log-rank test.

Serum albumin, prealbumin, total protein levels and total lymphocyte counts (TLC) were used for Pearson correlation analysis. The chi-square test and ANOVA were used to determine statistical significance. Statistical analyses were performed with SPSS 19.0 (SPSS Inc., Chicago, IL, USA). A two-tailed *P*-value of <0.05 was regarded as significant.

## Results

Between 2009 and 2012, 275 patients with glioblastoma were treated in our department. After the exclusion of patients as described above, 214 newly diagnosed patients were included in the final analysis, of whom 140 (65.4%) were under and 74 (34.6%) were over 60 years of age. In the KPS, 121 (56.5%) patients scored 70–100 and 93 (43.5%) scored less than 70. The mean preoperative serum albumin level was 35.63 ± 5.7 g/L (range 22.10–49.90 g/L). The mean follow-up period was 13.7 months (range 1–43 months), during which all patients died from glioblastoma. No patient was lost to follow-up. The median overall survival was 14.0 (95% CI 11.7 − 14.3) months. The corresponding 1- and 2-year survival rates were 60.3% (129/214) and 8.9% (19/214), respectively. In the CAG group, 2-year survival rate was 19.2% (15/78), which was less than the approximately 27% reported by Stupp et al. [1]. The heterogeneity of post-progression salvage treatment may result in the differences of surival rate. Clinicopathologic data are summarized in Table 1.

**Table 1:** **Clinical and molecular characteristics according to serum albumin levels in 214 glioblastoma cases**

Clinical or molecular feature	All cases			Serum albumin levels						*P*
				<30 g/L			≥30 g/L			
	No.		%	No.		%	No.		%	
**Total no. of patients**	214		100	28		13.1	186		86.9	
**Sex**										
Male	120		56.1	18		15	102		85	0.417
Female	94		43.9	10		10.6	84		89.4	
**Age, years**										
Mean ± SD		52.3 ± 12.8			52.3 ± 14.9			52.3 ± 12.6		0.995
**Tumor size, cm** ^**3**^										
Mean ± SD		62.9 ± 27.1			62.6 ± 26.8			63.0 ± 27.2		0.948
**KPS**										
Mean ± SD		66.4 ± 13.7			58.2 ± 14.9			67.6 ± 13.1		*0.001*
**Resection**										
Biopsy	15		7.0	2		13.3	13		86.7	0.960
Subtotal	102		47.7	14		13.7	88		86.3	
Gross total	97		45.3	12		12.4	85		87.6	
**Adjuvant treatment**										
NAG	58		27.1	13		22.4	45		77.6	*0.004*
PAG	77		36.0	12		15.6	65		84.4	
CAG	79		36.9	3		3.8	76		96.2	
**MGMT promoter**										
Methylated	99		46.3	16		16.2	83		83.8	0.229
Unmethylated	115		53.7	12		10.4	103		89.6	
**IDH1** ^**R132H**^ **mutation**										
Positive	14		6.5	3		21.4	11		78.6	0.338
Negative	200		93.5	25		12.5	175		87.5	
**Prealbumin, mg/L**										
Mean ± SD		247.0 ± 51.2			224.1 ± 36.7			250.5 ± 52.2		*0.011*
**Total protein, g/L**										
Mean ± SD		61.2 ± 7.0			58.8 ± 5.7			61.6 ± 7.1		0.052
**TLC, 10** ^**9**^ **/L**										
Mean ± SD		1.8 ± 0.8			1.7 ± 0.8			1.8 ± 0.7		0.302

CAG: completely applied group; IDH, isocitrate dehydrogenase; KPS: Karnofsky Performance Scores; MGMT: O(6)-methylguanine-DNA-methyltransferase; NAG: not applied group; PAG: partially applied group; TLC: total lymphocyte counts.

**Figure 1:**
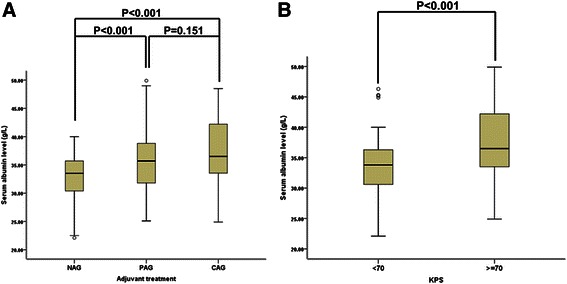
**Correlation between preoperative serum albumin levels and other clinical factors in glioblastoma patients. (A)** Relationship between preoperative serum albumin levels and adjuvant treatment. For adjuvant treatment: CAG, completely applied group; NAG, not applied group; PAG, partially applied group. **(B)** Relationship between preoperative serum albumin levels and KPS.

In this study, serum albumin level was significantly correlated with adjuvant treatment and KPS. As shown in Figure 1A, the serum albumin levels of patients in the CAG and PAG groups (mean ± SD: 37.4 ± 5.6 and 36.1 ± 6.0 g/L, respectively) were markedly higher than that of patients in the NAG group (32.6 ± 4.3 g/L; *P* < 0.001). Moreover, serum albumin levels in patients with KPS >70 (37.4 ± 4.8 g/L) were remarkably higher than those in patients with KPS <70 (33.4 ± 4.9 g/L; *P* < 0.001) (Figure 1B). Serum albumin levels did not vary significantly with sex, age, tumor size, degree of resection, MGMT promoter methylation and IDH1-R132H mutation status.

### Serum albumin level and the survival of glioblastoma patients

**Figure 2:**
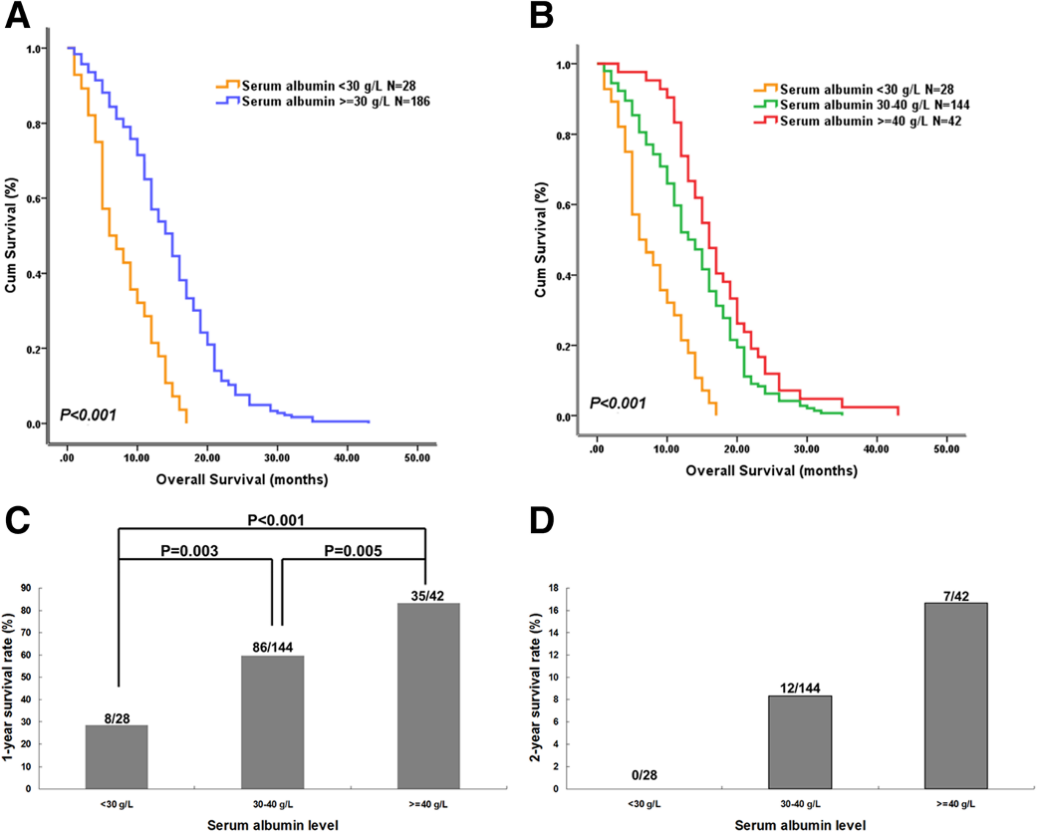
**Preoperative serum albumin levels and prognosis. (A, B)** Kaplan–Meier survival curves stratified by preoperative serum albumin levels. Survival was significantly lower among patients with low serum albumin (<30 g/L) than in those in the normal range (≥30 g/L; **A)**. Patients with upper normal albumin levels (≥40 g/L) experienced longer survival than patients with lower normal albumin levels (30–40 g/L; **B)**. **(C, D)** Preoperative serum albumin levels were correlated with 1-year **(C)** and 2-year **(D)** survival rates.

Next, we examined the survival function of preoperative serum albumin levels in glioblastoma patients. Univariate and multivariate Cox regression analyses showed that serum albumin level was an independent predictor of OS (multivariate HR = 0.966, 95% CI 0.938-0.995, *P* = 0.023, Table 2). Patients with low serum albumin levels (<30 g/L) had a significantly shorter overall survival than those with levels in the normal range (median 6.0 vs. 15.0 months, log-rank test *P* < 0.001; Figure 2A). Moreover, the OS of patients with albumin in the upper normal range (≥40 g/L) was also longer than that of patients with albumin in the lower normal range (30–40 g/L; median 16.0 vs. 13.0 months; Figure 2B). As shown in Figure 2C and D, the 1-year and 2-year survival rates increased with preoperative serum albumin level.

**Table 2:** **Univariate and multivariate analyses of different prognostic parameters for overall survival of 214 glioblastoma patients**

Variable	Univariate			Multivariate		
	*P*	HR	95% CI	*P*	HR	95% CI
Sex	0.880	1.021	0.778-1.339	0.266	1.176	0.883-1.566
Age	0.059	1.011	1.000-1.022	0.466	1.004	0.993-1.016
Tumor size	0.474	1.002	0.997-1.007	0.381	1.002	0.997-1.008
KPS	<0.001	0.964	0.954-0.974	<0.001	0.974	0.963-0.986
Resection	0.014	0.731	0.568-0.940	0.091	0.800	0.618-1.036
Adjuvant treatment	<0.001	0.577	0.487-0.684	0.001	0.726	0.597-0.884
MGMT promoter	0.162	0.823	0.626-1.081	0.925	1.015	0.749-1.375
IDH1-R132H mutation	0.006	0.450	0.255-0.794	0.018	0.489	0.270-0.885
Serum albumin level	<0.001	0.938	0.912-0.964	0.023	0.966	0.938-0.995

IDH, isocitrate dehydrogenase; KPS: Karnofsky Performance Scores; MGMT: O(6)-methylguanine-DNA-methyltransferase.

**Figure 3:**
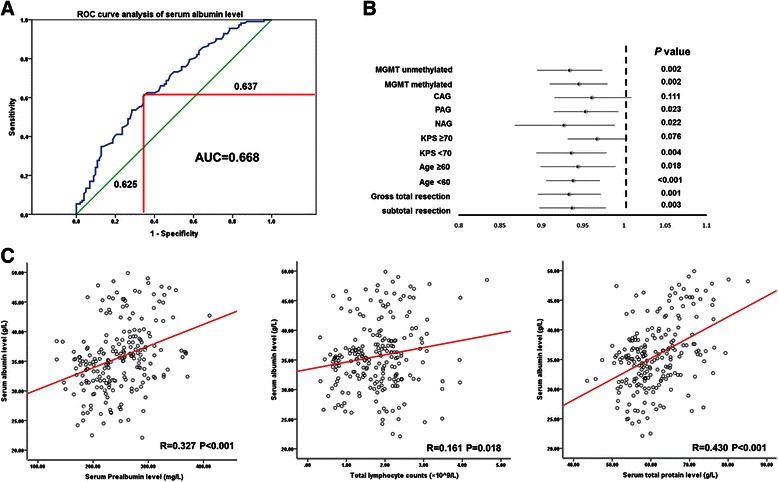
**Receiver-operator characteristic (ROC) curve and stratified analysis. (A)** ROC curve analysis of preoperative serum albumin level. In 214 glioblastoma patients, at the median level (35.35 g/L) of preoperative serum albumin, the discriminative power reached 62.5% sensitivity and 63.7% specificity for short-survival cases (<1 year) versus long-survival cases (>1 year). AUC, area under the curve. **(B)** Stratified analysis of preoperative serum albumin level and overall mortality. Hazard ratios and 95% confidence intervals in various strata are shown. For adjuvant treatment: CAG, completely applied group; NAG, not applied group; PAG, partially applied group. **(C)** Preoperatively, serum albumin levels were significantly correlated with prealbumin, total protein levels and total lymphocyte counts.

All 214 glioblastoma cases were used to construct a ROC curve to assess the prognostic performance of preoperative serum albumin level for glioblastoma patients. We used 1 year as a time horizon. Patients with OS longer than 1 year were designated as long-survival cases, and those with OS shorter than 1 year as short-survival cases. According to the ROC curve, at the median preoperative serum albumin level (35.35 g/L), the discriminative power was 63.7% specificity and 62.5% sensitivity (Figure 3A).

Multivariate analysis showed that KPS, adjuvant treatment and IDH1-R132H mutation were independently associated with OS in glioblastoma (Table 2). Among patients who received complete adjuvant treatment (CAG), survival was significantly longer in patients with MGMT promoter methylation (multivariate HR = 0.618, 95% CI 0.387–0.988, *P* = 0.044), which was consistent with the results of previous studies and widely accepted by other researchers. However, in patients who did not receive adjuvant treatment or did not complete the treatment protocol (NAG and PAG), MGMT methylation status was not a prognostic factor. Thus, high MGMT promoter methylation predicted a good outcome only when patients received complete adjuvant therapy.

### Stratified analysis of serum albumin level and prognosis

We further examined the influence of preoperative serum albumin levels on OS across strata of other potential predictors, including age, preoperative KPS, degree of resection, MGMT promoter methylation status and adjuvant treatment. The number of cases with IDH1 mutation was too small to be included in the stratified analysis. Serum albumin level was an independent predictor in most of the subgroups except in patients who received complete adjuvant treatment (Figure 3B). In CAG, the preoperative albumin levels were high (mean ± SD: 37.4 ± 5.6 g/L) and only three patients with albumin levels lower than 30 g/L, which may affect the prognostic significance of albumin levels.

### Serum albumin and other nutritional indicators

In this study, we found that preoperatively, serum albumin levels significantly correlated with other nutritional indicators, including serum prealbumin, total protein levels and total lymphocyte counts in glioblastoma patients (Table 1 and Figure 3C). In univariate analysis, these factors were all associated with patient survival. However, in multivariate analysis, the prognostic significance of other nutritional indicators was markedly diminished by serum albumin level (Table 3). Meanwhile, as another nutritional indicator, hemoglobin level did not correlate with albumin level and was not associated with patient survival in this study (data not shown).

**Table 3:** **Univariate and multivariate analyses of different nutritional indicators for overall survival of 214 glioblastoma patients**

Nutritional indicators	Univariate			Multivariate		
	*P*	HR	95% CI	*P*	HR	95% CI
Serum prealbumin level	0.003	0.996	0.993-0.999	0.041	0.997	0.993-1.000
Serum total protein level	0.029	0.978	0.960-0.998	0.485	1.009	0.985-1.033
Total lymphocyte counts	0.035	0.818	0.678-0.986	0.072	0.841	0.696-1.015
Serum albumin level	<0.001	0.938	0.912-0.964	<0.001	0.942	0.914-0.972

## Discussion

Identification of prognostic factors is clinically relevant for glioblastoma patients and can guide clinical treatment and studies [13]. Preoperative serum albumin levels have been recognized as prognostic in glioblastomas [8,9]. However, this prognostic effect should be re-examined in the era of standard therapy [1], when molecular markers such as MGMT promoter methylation status are taken into consideration [14,15]. Moreover, the mechanism by which serum albumin levels can predict the prognosis of glioblastoma patients should be further explored.

**Figure 4:**
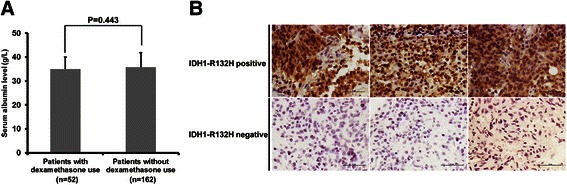
**Immunohistochemistry for IDH1-R132H mutation. (A)** Serum albumin levels in patients with or without dexamethasone use. **(B)** Representative immunohistochemical images of tumors with or without IDH1-R132H mutation (×400). Scale bar: 50 μm.

In the present study, we found that preoperative serum albumin levels significantly correlated with survival in glioblastoma patients who received no or incomplete adjuvant treatment. However, in patients who received complete adjuvant treatment, the correlation between serum albumin levels and survival was insignificant (Figure 3B). Our data showed that in CAG, the patients’ serum albumin levels were high, and very few patients had hypoalbuminemia. This phenomenon may reflect the fact that patients who were in good general condition were more likely to complete the adjuvant treatment than patients who were not. The overall high level may affect the prognostic significance of serum albumin. Nevertheless, we cannot rule out the possibility that serum albumin level was not a potential predictor in patients who received complete adjuvant treatment for unclear reasons, and stronger conclusion should be drawn in future studies including a larger number of hypoalbuminemia cases who complete standard therapy. Totally, 52 (24.3%) cases used dexamethasone prior to the serum albumin measurement for only one day with a total dose of 5–10 mg. The remaining cases received no steroid before the measurement. The serum albumin levels were similar for patients with and without dexamethasone use (35 ± 5.0 vs 35.8 ± 6.0 g/L, *P* = 0.443; Figure 4A), consistent with previous data that a short time and small dose of steroid therapy is unlikely to affect the serum albumin level [16]. Although IDH mutation is associated with a better prognosis, only 5- 10% of individuals with adult glioblastoma carry an IDH mutation [13]. IDH1- R132H mutation accounts for nearly 90% of all IDH mutations and can be demonstrated by immunohistochemistry, although other mutations can only be identified by sequencing [11,13]. In this series of cases, immunohistochemistry identified IDH1-R132H mutation in 14 (6.5%) tumors (Figure 4B), which was not associated with serum albumin level (Table 1). Moreover, the prognostic effect of serum albumin levels was not significantly modified by MGMT promoter methylation status, suggesting that these factors influence clinical outcome via different pathways.

The role of serum albumin as a nutritional indicator is well established, although other markers such as prealbumin are more sensitive [17,18]. In this study, we found that in association with the nutritional status of patients, serum albumin levels correlated with prealbumin, total protein levels and total lymphocyte counts as well as KPS (Figure 1B and Figure 3C). In univariate analysis, all these nutritional indicators were associated with OS, which demonstrated the prognostic value of nutritional indicators (Table 3). Thus, the prognostic effect of serum albumin level can be at least partly attributed to its role as a nutritional indicator. However, in multivariate analysis, the prognostic significance of prealbumin, total protein levels and total lymphocyte counts was markedly reduced by serum albumin levels. Consistent with our results, previous studies reported that serum albumin levels carried a greater prognostic value than other nutritional indicators in patients with various malignancies [19,20]. In our opinion, this is not because serum albumin is a stronger indicator of nutritional status, but rather because it is involved in specific pathophysiological processes such as inflammatory responses.

In our previous study, we demonstrated that serum IGFBP-2 levels were inversely correlated with survival in glioblastoma patients [5]. The underlying mechanism may be that a high concentration of exogenous IGFBP-2, possibly resulting from blood–brain barrier (BBB) leakage, stimulates proliferation, invasion, and chemoresistance to temozolomide in glioblastoma cells via the integrin β1-ERK pathway [21]. In the present study, we showed that serum albumin level was positively correlated with survival in glioblastoma patients. Low serum albumin levels have been shown to be significantly associated with higher IGFBP-2 levels in many pathophysiological conditions [6,22,23]. Moreover, the permeability of BBB may be greater among glioblastoma patients with low serum albumin levels [8]. Thus, low serum albumin levels associated with high serum IGFBP-2 levels and BBB leakage may result in poor survival.

In addition, tumor cells can induce inflammatory responses [9]. In inflammatory conditions, high serum IGFBP-2 levels are associated with elevated cytokine interleukin (IL)-6 [22,24-26], another prognostic factor for glioblastoma [9,27] that negatively regulates serum albumin level by increasing catabolism and down-regulating hepatic synthesis, which further worsens the nutritional status of the patient. Therefore, during glioblastoma-induced inflammatory responses, the interaction among albumin, IGFBP-2 and IL-6 may greatly affect clinical outcomes. The levels of serum albumin may reflect the severity of the inflammatory reaction and the patient’s general condition, thus predicting survival.

The present study had several limitations. First, the retrospective design of the study may lead to bias. Second, the lack of serial dynamic serum albumin levels is another limitation. Third, the number of cases who completed standard therapy was not large enough and their serum albumin levels were high, which limits the power of this study. Prospective data collection in a larger sample should be performed when possible to achieve stronger conclusions.

## Conclusion

We showed that serum albumin level is associated with prognosis in glioblastoma patients. Further investigation including a larger number of cases with various levels of serum albumin who received complete standard therapy would validate this result. The mechanism by which serum albumin levels predict clinical outcome is complex, not only because it is a nutritional indicator, but also because of its role in the inflammatory response.

## Abbreviations

BBB: Blood–brain barrier
CAG: Completely applied group
HR: Hazard ratio
IDH: Isocitrate dehydrogenase
IGFBP-2: Insulin-like growth factor-binding protein-2
KPS: Karnofsky performance status
MGMT: O-methylguanine-DNA methyltransferase
MSMLPA: Methylation-specific multiplex ligation-dependent probe amplification
NAG: Not applied group
OS: Overall survival
PAG: Partially applied group
TLC: Total lymphocyte counts
TMZ: Temozolomide
